# Mechanisms of Macrophage Plasticity in the Tumor Environment: Manipulating Activation State to Improve Outcomes

**DOI:** 10.3389/fimmu.2021.642285

**Published:** 2021-05-07

**Authors:** Tiffany Davia Ricketts, Nestor Prieto-Dominguez, Pramod Sreerama Gowda, Eric Ubil

**Affiliations:** Department of Microbiology, University of Alabama at Birmingham, Birmingham, AL, United States

**Keywords:** cancer, macrophage, plasticity, therapy, tumor, inflammation

## Abstract

Macrophages are a specialized class of innate immune cells with multifaceted roles in modulation of the inflammatory response, homeostasis, and wound healing. While developmentally derived or originating from circulating monocytes, naïve macrophages can adopt a spectrum of context-dependent activation states ranging from pro-inflammatory (classically activated, M1) to pro-wound healing (alternatively activated, M2). Tumors are known to exploit macrophage polarization states to foster a tumor-permissive milieu, particularly by skewing macrophages toward a pro-tumor (M2) phenotype. These pro-tumoral macrophages can support cancer progression by several mechanisms including immune suppression, growth factor production, promotion of angiogenesis and tissue remodeling. By preventing the adoption of this pro-tumor phenotype or reprogramming these macrophages to a more pro-inflammatory state, it may be possible to inhibit tumor growth. Here, we describe types of tumor-derived signaling that facilitate macrophage reprogramming, including paracrine signaling and activation of innate immune checkpoints. We also describe intervention strategies targeting macrophage plasticity to limit disease progression and address their implications in cancer chemo- and immunotherapy.

## Introduction

Macrophages represent one of the most phenotypically diverse innate immune cell populations. They are key homeostatic regulators that activate and modulate the innate and, subsequent adaptive immune response to infectious agents and host-derived components. Much like other innate immune cells, they are hard-wired to respond to cues rather than being “educated” to elicit a response, as is the case of adaptive immune cells (1). Macrophages are equipped with a variety of Pattern Recognition Receptors (PRRs) that, once activated, trigger pre-determined programs in response to environmental stimuli. Some pro-inflammatory stimuli include Pathogen-Associated Molecular Patterns (PAMPs), cellular or chemical moieties derived from pathogens, or Damage-Associated Molecular Patterns (DAMPs) which are released by damaged cells and malignancies. These signatures permit macrophage adoption of the appropriate functional phenotype to restore physiological equilibrium.

**Figure 1:**
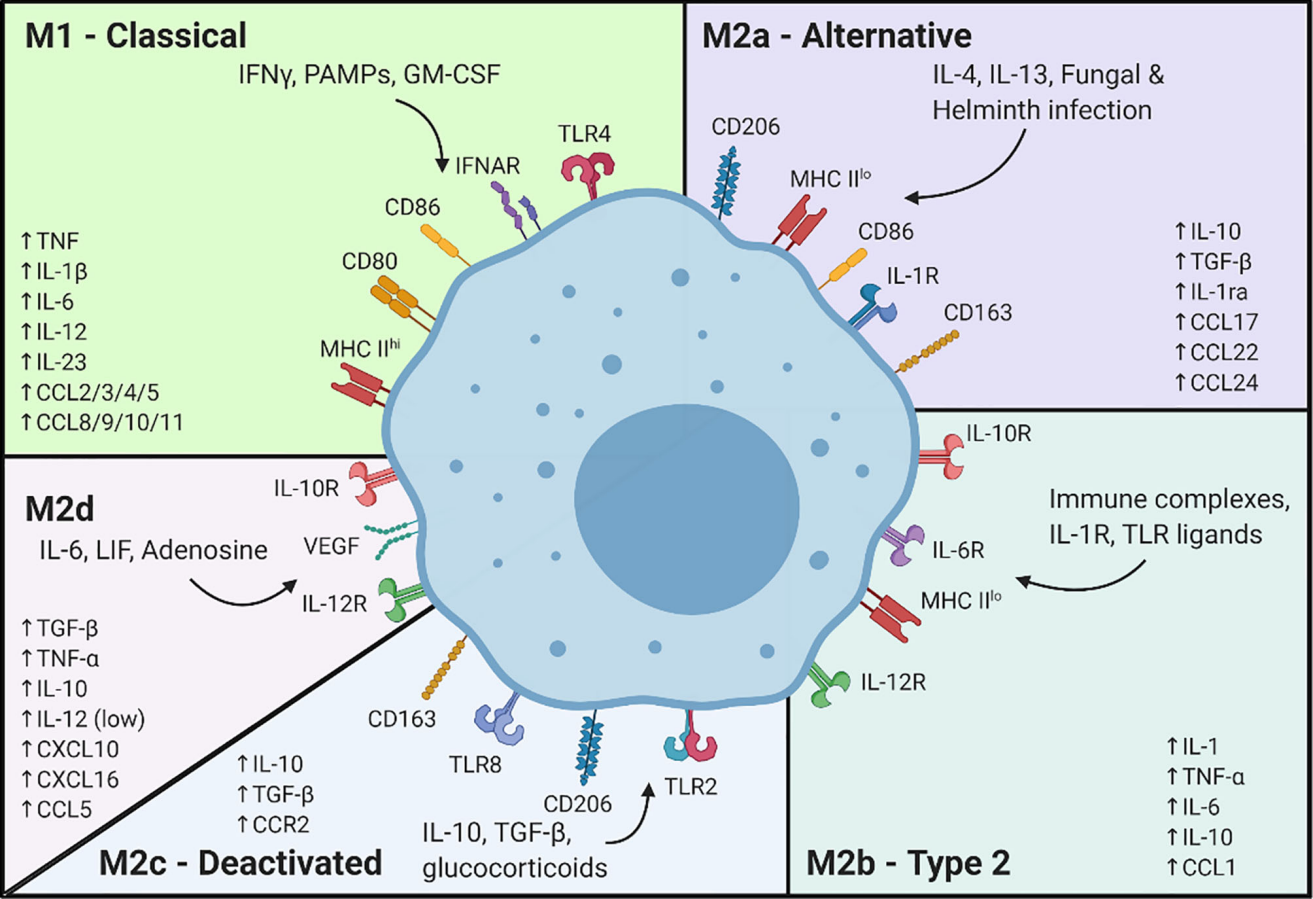
Signals associated with macrophage differentiation to the classically and alternatively activated subsets. Created with BioRender.

**Figure 2:**
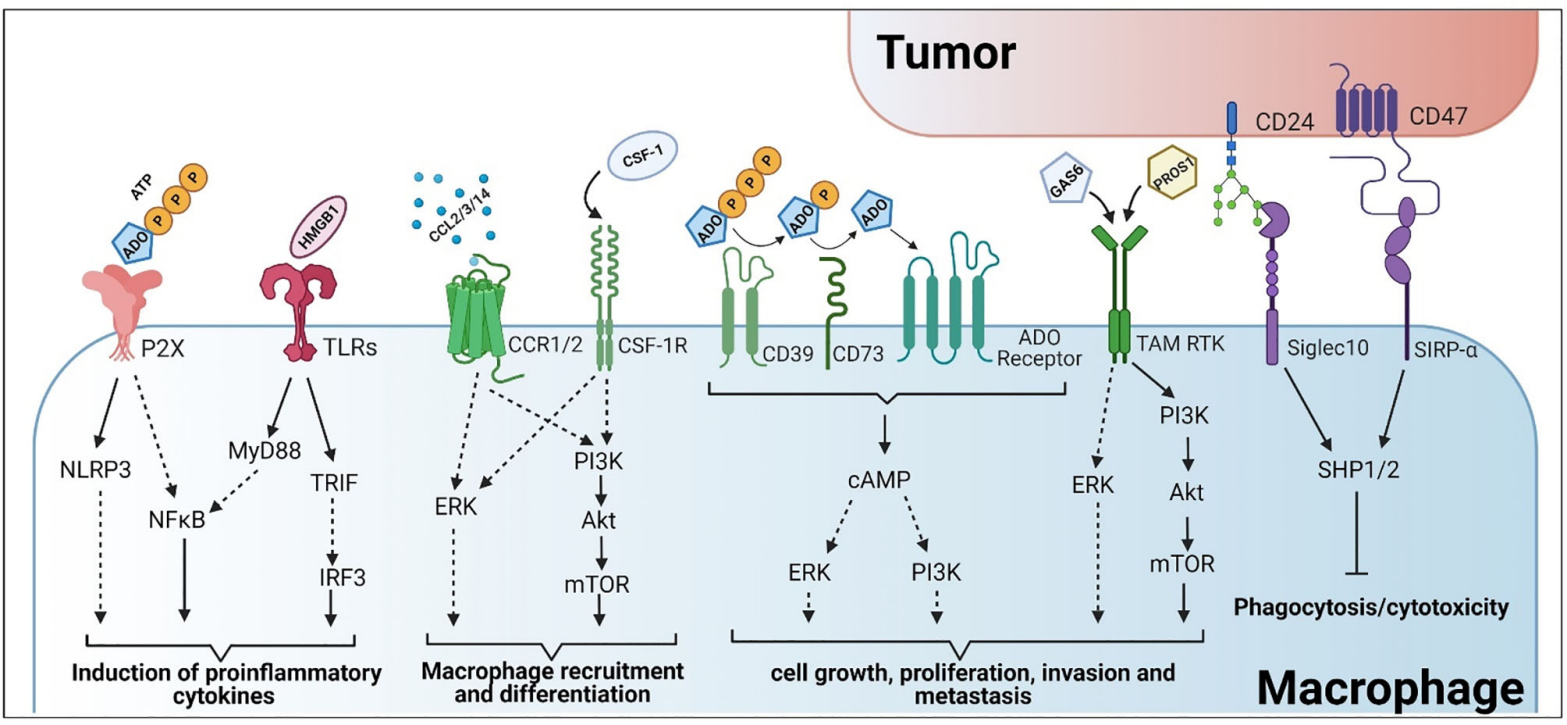
Tumor-macrophage interactions and their subsequent roles in immune evasion and activation. Created with BioRender.

During infections, macrophage polarization to the proinflammatory state is crucial for the production of type 1 cytokines such as interferon-γ (IFNγ), tumor necrosis factor-α (TNFα) and interleukin 12 (IL-12) for host resistance (2–4). This is similar to the response following injury. Cells in damaged tissues undergo necrosis and release their contents in an uncontrolled manner (5–7). Contrary to apoptosis, which is a highly organized program for cell death, necrosis is more immunogenic and induces a macrophage pro-inflammatory response. Cellular components released during necrosis act as DAMPs that, when bound to PRRs like Toll-like Receptors (TLRs), initiate pro-inflammatory signaling in resident and extravasated monocyte-derived macrophages. Activation of PRRs, and other sensors, facilitate the adoption of a pre-programmed pro-inflammatory state, also termed M1 or “classically activated” (**Figure 1**). This occurs through increased activation of signaling pathways involving NFκB, p38, MAPK, and others, which regulate the expression of pro-inflammatory cytokines (e.g., IL-1, IL-6, IL-12 (8, 9)) (**Figure 2**). These macrophage-secreted signals recruit a variety of other immune cells that pioneer the clearance of infected and damaged material.

A hallmark of the pro-inflammatory response is the destruction of damaged cells and those in the immediate vicinity. This creates a need for wound healing to restore tissue integrity. Upon removal of damaged tissue, the aggregate population of macrophages at the site of injury transitions to a pro-wound healing phenotype, also referred to as M2 (**Figure 1**). This transition is triggered by anti-inflammatory mediators following the loss of pro-inflammatory signals, like DAMPs. These pro-wound healing macrophages coordinate the proliferation of key cell types including vascular endothelial cells, which promote recellularization by delivering oxygen and nutrients to the site of repair, and fibroblasts which drive scar formation (10–12). Macrophages also dampen the local inflammatory response, fostering a more hospitable environment for continued repair, cellular proliferation and the prevention of extensive or persistent inflammation that might contribute to further tissue damage (13–16).

While macrophage plasticity is beneficial during the wound healing process, the macrophage response is subverted during cancer. Often termed “a wound that does not heal” (17), tumors manipulate and reshape the immune response to promote and sustain tumor growth. Presumably, due to the inhospitable nature of the tumor microenvironment (e.g., hypoxia, nutrient starvation), cancer cells undergo necrotic death which should induce the macrophage pro-inflammatory response, ultimately leading to further immune activation and reduced tumor growth. However, in many tumors, the pro-wound healing phenotype is predominant, which actually supports cancer progression. This review outlines strategies employed by tumors to mitigate macrophage pro-inflammatory activation or engage the pro-wound healing response. Current therapeutic interventions that alter the intra-tumoral M1/M2 balance and shift it towards a more pro-inflammatory/anti-tumor response are also described. We also explore potential conceptual flaws in the current pro-inflammatory/pro-wound healing paradigm in cancer, based on recent single-cell RNA-seq findings, and implications these could have in the manipulation of macrophage activation state to reduce tumor growth.

## The Role of Macrophages in the Anti-Tumor Response

During tumorigenesis, genetic mutations can be acquired through exposure to chemical carcinogens (18), radiation (19) or viral infections (20, 21). Alternatively, inherited mutations (22, 23) or those accumulated during chronic inflammation (24–26) may also drive carcinogenesis. Cell intrinsic tumor suppressive mechanisms, like DNA repair, senescence or apoptosis (27), often fail to contain tumor cell proliferation, promoting the need for immune-mediated elimination of the aberrant cells. Ideally, early responding immune cells, like macrophages, will detect and eliminate tumor cells. Much like during wound healing, macrophages may detect DAMPs, possibly from hypoxia-induced tumor cell death or dysregulated cellular processes (28), to trigger a pro-inflammatory response and pave the way for true wound healing or a return to homeostasis. Alternatively, macrophages or dendritic cells, as antigen presenting cells, may engulf tumor neo-antigens, process them and present antigenic peptides to tissue resident CD8+ or CD4+ T cells, or in the case of dendritic cells, transit to the draining lymph node to activate T cells (29–31). Whether for tissue resident or T cells transiting from the lymph nodes, pro-inflammatory macrophages provide co-stimulatory signals such as CD40 (32) or CD80/86 (33), secrete activating cytokines (34), and generate nitric oxide to increase vascular permeability and immune cell infiltrate. T cells with the cognate receptor matching the tumor neo-antigen, in the presence of co-stimulation, should eradicate tumor cells unless they encounter other immuno-suppressive signals.

While many early-stage tumors are presumably destroyed through these mechanisms, the immune response to cancer is clearly not effective. Rather, based on the immune-editing hypothesis (35), the pro-inflammatory response applies a selective pressure, forcing tumors to “evolve” to avoid detection (e.g., through reduced antigenic protein expression, reduction in antigen presentation (35) or suppression of the local immune response (36)). Alternatively, nascent tumors may undergo a period of dormancy, and may later be reactivated by acquired secondary or tertiary mutations that allow for reduced immunogenicity or increased immune suppression. Collectively, this evolution is thought to allow tumor cells to reach an equilibrium with the immune response. Following this equilibrium state, tumors may effectively “escape” the immune response by utilizing mechanisms to prevent immune activation, allowing them to grow largely unchecked.

Consequently, these immuno-editing processes may limit macrophage responsiveness to DAMPs and tumor neo-antigens, effectively abrogating their ability to transition to an M1 phenotype (37) and promote T cell activation. In many tumors, there is a promotion of the M2 phenotype which fosters tumor growth. Presumably, either acquired through the equilibrium/escape processes of immuno-editing or because tumors provide contextual cues similar to those that promote the pro-wound healing response. These M2 macrophages are pro-tumorigenic and are often denoted as tumor-associated macrophages (TAMs). Akin to the wound healing response, macrophages facilitate cellular proliferation through production of growth factors like Wnts (38), CXCL8 (39) or IL-6 (40, 41). However, instead of promoting the re-growth of tissue resident cells, these factors drive tumor growth. Likewise, macrophages also secrete key effectors of vascularization, like the vascular endothelial growth factor (VEGF) (42, 43), platelet-derived growth factor (PDGF) (44) and transforming growth factor β (TGFβ) (45) to promote angiogenesis (**Figure 1**). These physiologic processes are hijacked to increase blood flow to the tumor, increasing tumor cell access to oxygen and nutrients for continued cell proliferation. M2 macrophages may also maintain tumor growth through the remodeling of the extracellular matrix (ECM) through secretion of matrix metalloproteases (MMPs) and other factors (45, 46) (**Figure 1**).

In the tumor context, pro-inflammatory macrophages are considered a positive prognostic marker (47–49). Pro-inflammatory macrophages are thought to positively regulate the immune response and kill tumor cells directly. These polarized macrophages prevent tumor growth by generating factors such as reactive oxygen and nitrogen species, or other secreted factors like TNFα, that lead to tumor cell death (50–53). Macrophages can be induced to a pro-inflammatory state by other immune cells, such as through the secretion of IFNγ by T cells, or directly by tumor cells. Alternatively, DAMPs can be released by necrotic or necroptotic tumor cell death due to hypoxia or nutrient deprivation within the tumor microenvironment (54, 55). These DAMPs, whether they be nucleic acids, ATP, stress-related proteins such as heat shock proteins (HSPs) (56–58), or transcription factors such as HMGB1, HMGN1 (59–65), bind to and activate two major classes of PRRs including the TLRs or the NOD-like receptor (NLR) family. Interestingly, several TLRs that recognize pathogenic signatures also recognize DAMPs. For instance, TLR4, which is activated by the binding of bacterial lipopolysaccharide (LPS) also recognizes HSPs and transcription factors (66).

Conversely, the presence of M2 pro-wound healing macrophages in tumors is generally a negative prognostic marker, with patients with high numbers of intra-tumoral M2 macrophages showing decreased survival (67). Tumor cells are known to secrete, or induce the secretion of, factors like IL-4, IL-10 or IL-13 that polarize macrophages toward an M2 phenotype (44, 68). Some pro-wound healing properties of M2 macrophages foster tumor growth and prepare a tumor-friendly milieu (**Figure 1**). M2 macrophages can act to directly increase tumor growth by secretion of growth factors like endothelial growth factor (EGF), VEGF and TGFβ (69–73), and can reduce the hypoxia inherent in most tumors while allowing the delivery of nutrients to sustain tumor growth. M2 macrophages also assist in the remodeling of the tumor microenvironment. Regulation of fibroblast ECM placement, degradation of existing ECMs through MMPs and chemotactic migration signals, allow continued tumor growth and metastasis. In some cases, live cell imaging has shown tumor cells utilizing accessory macrophages to travel to blood vessels and allow entry into the vasculature (74–76).

## Macrophage-Directed Therapeutic Strategies for Cancer Treatment

Based on knowledge garnered from the study of macrophage activation states in tumors, as well as associated signaling affecting polarization, several strategies have been developed to mitigate tumor progression by altering macrophage infiltration or by activating/re-activating them to a pro-inflammatory state. While a limited number of macrophage-directed therapeutics are currently in use in clinical trials, continued identification and pharmacological targeting of macrophages is expected to bolster the use of macrophage targeted agents.

### Macrophage Depletion to Reduce Pro-Tumoral Activity

Since higher numbers of TAMs are associated with worse cancer prognosis, research has focused on reducing their numbers by targeting their tumor recruitment and differentiation (77–79). As a result, some of the subsequent strategies are being tested for clinical use and may be broadly available soon.

Macrophages, similar to other phagocytes, can be selectively targeted by complexing cellular pro-apoptotic substances, such as bisphosphonates, into nanoparticles (80) (**Table 1**). The deletion of TAMs by using clodronate encapsulated in liposomes (clodrolip) leads to reduced teratocarcinoma and rhabdomyosarcoma tumor growth in pre-clinical murine studies (144). This inhibition was coupled with a decrease in tumor microvascular density, suggesting its potential combination with VEGF-neutralizing agents to maximize its effect (144).

**Table 1:** Summary of preclinical, clinical and current therapeutic approaches targeting macrophages for the treatment of various malignancies.

Therapeutic Agent	Therapeutic Modality	Indication	Target	Effect	Development Status	References
Anti-CCR2	Monoclonal antibodies (mAbs), small molecule inhibitor	Metastatic solid tumors	CCL2/CCR2	CCR2 antagonist blocks the adaptation of TAM features	Phase I/II clinical trials	(81–84)
Anti-CD24	mAbs	Advanced solid tumors	CD24/Siglec10	Increases expression of M1 macrophages and phagocytosis	Preclinical	(85, 86)
Anti-CD39	mAbs	Advanced solid tumors	CD39	Increases extracellular ATP, promotes M1 phenotype	Phase I clinical trials	(87–89)
Anti-CD40	Vaccine, mAbs	Lung cancer, metastatic melanoma, solid cancers	CD40	CD40 agonism promotes proinflammatory activity and increases antigen presentation	Phase I/II clinical trials	(90–93)
Anti-CD47	mAbs	Advanced solid tumors, hematologic malignancies	CD47/SIRPa	Increases macrophage phagocytosis and M1 activation	Phase I/II clinical trials	(94–96)
Anti-CD73	mAbs	Advanced or metastatic cancer	CD73	Promotes anti-tumorigenic macrophage activation	Phase I/II clinical trials	(87, 88, 97)
Anti-CSFR1	Blocking antibodies, small molecule inhibitor (BLZ945)	Advanced solid tumors	CSF1/CSFR1	Increases proinflammatory and tumoricidal activity, inhibits recruitment of immunosuppressive populations	Phase I/II clinical trials	(98–101)
Bemcentinib	Small molecule inhibitor	Advanced or Metastatic Solid Tumors	Axl RTK	Inhibits polarization to the anti-inflammatory macrophage phenotype	Phase I/II clinical trials	(102–104)
BMS-777607	Small molecule inhibitor	Advanced solid tumors	TAM RTKs	Restores proinflammatory immune activation, decreases immune suppressive cytokines and efferocytosis	Phase I/II clinical trials	(105, 106)
Clodronate	Bisphosphonate	Breast, prostate and bone neoplasms	Complement receptors	Depletes TAMs	Phase III	(107–111)
CpG ODN	Single stranded DNA, vaccine adjuvant	Breast cancer, malignant melanoma, glioblastoma, leukemia	TLR9	TLR9 agonist to switch macrophage polarization to proinflammatory	Phase I/II clinical trials	(112–114)
Dasatinib	Small molecule inhibitor	Chronic myeloid leukemia (CML), acute lymphocytic leukemia (ALL) advanced cancer	Src family tyrosine kinases	TAM depletion	Phase IV clinical trials, FDA approved for CML and ALL	(115–117)
Ferumoxytol	Metallic nanoparticles	Breast cancer, small cell lung cancer	Varies based on surface conjugates of nanoparticles	Reprograming of TAMs to tumoricidal, proinflammatory macrophages	Pre-clinical	(118–120)
IL-12	Polymeric nanoparticles, vaccine, gene therapy	Metastatic cancer, solid tumors	IL-12R	Re-education of TAMs	Phase I/II clinical trials	(121. 122, 123)
Imatinib	Small molecule inhibitor	Metastatic, advanced solid tumors, refractory malignancies	STAT6	Inhibits macrophage polarization to anti-inflammatory subset	Phase IV clinical trials FDA approved for CML	(80, 124, 125)
Imiquimod	Topical, vaccine, small molecule inhibitor	Basal cell carcinoma (BCC), skin cancer, solid tumors	TLR7	Reprogramming TAMs toward proinflammatory phenotype	Phase IV clinical trials	(126–128)
Nilotinib	Small molecule inhibitor	Solid tumors, neoplasms, gastrointestinal stromal tumors	BCR-ABL	Inhibits macrophage polarization to anti-inflammatory subset	Phase IV clinical trials FDA approved for CML	(80, 125)
P2X7 antagonism	Topical	BCC	ATP/purinergic receptor	Promotes M1 activation and phagocytosis	Phase I	(129–131)
STAT3 Inhibitors	Small molecular inhibitor	Advanced solid tumors	STAT3	Inhibits polarization to anti-inflammatory phenotype	Phase I/II clinical trials	(132–134)
STAT6 inhibitors	Small molecular inhibitor	–	STAT6	Inhibits polarization to anti-inflammatory phenotype	–	(135–137)
Sunitinib	Small molecular inhibitor	Refractory solid tumors, renal cell carcinoma (RCC), gastrointestinal stromal tumors (GIST)	Multi-targeted RTKs	Blockade of anti-inflammatory phenotype	Phase IV clinical trials, FDA approved for RCC and GIST	(80, 138)
Zoledronic acid	Bisphosphonate	Breast cancer, prostate cancer, metastatic neoplasms	TLR4	Phenotype switch to proinflammatory	Phase IV clinical trials	(139–143)

Alternatively, inhibition of the chemotactic axis CCL2-CCR2 may prevent the accumulation of circulating macrophages within the tumor microenvironment. Indeed, several monotherapy or combinational clinical trials are currently underway with positive results (81). However, CCL2-CCR2 inhibitors should be carefully administered since the sudden interruption of therapeutic regimens could dramatically increase tumor progression and metastasis (145).

Additionally, targeting the monocyte/macrophage colony stimulating factor (CSF-1) and its receptor (CSF-1R) is a tractable strategy for macrophage depletion. In the absence of this signal, bloodborne monocytes are unable to differentiate into macrophages, preventing macrophage tumoral accumulation (146). Accordingly, several CSF-1R/CSF-1 targeted therapies, such as PLX3397, JNJ-40346527 and BLZ945, are currently being tested in clinical trials either alone or in combination for the treatment of several cancers (98, 147–149). However, these inhibitors can also stimulate the recruitment of tumor-promoting granulocytes to the site of the tumor, resulting in therapy failure (150). Therefore, combination of CSF-1R repressor with adaptive immune checkpoint inhibitors may be an interesting strategy to mitigate this unexpected effect (150).

Finally, the antineoplastic agent, trabectedin, also depletes TAMs to induce pro-inflammatory T cell recruitment in pancreatic ductal adenocarcinoma preclinical models (151). Therefore, it could also be a potential new strategy for TAM depletion during cancer treatment.

### Manipulating Macrophage Activation State to Improve the Anti-Tumor Response

Using *in vitro* models of macrophage polarization, it has been shown that responses to respective M1/M2 stimuli are transient. Treatment with M1 inducing agents, like LPS and IFNγ, induce a pro-inflammatory response within 2-4 hours, which may subside within 24-48 hours (51, 152). After this transient activation, macrophages return to a “resting” state akin to the naïve (M0) polarization. Likewise, activation with one stimulus does not preclude the ability to adopt a subsequent, alternative polarization. A notable example is when stimulating conditions are switched from IFNγ to IL-4 or vice versa, macrophages adopt the profile of the most current cytokine microenvironment (153). Gao and colleagues utilized M-CSF and IL-4 to induce human monocyte differentiation to the M2 phenotype. Following M2 polarization, macrophages were treated with lactoferrin-containing IgG immunocomplex (LTF-IC), which promotes M1-like activation and is an immune activator in rheumatoid arthritis (154). After M1 stimulation, M2 marker expression was reduced while M1 markers were increased. In a similar experiment, Cheng et al. induced M2 polarization in murine RAW264.7 cells using IL-4 and IL-13. Subsequent treatment of M2 macrophages with a β-1,6-glucan (AAMP-A70) caused a reduction of M2 polarization concurrently with increased M1 marker expression (155). These findings are particularly important in the context of cancer treatment, as they clearly demonstrate the plasticity of macrophages depend on the environmental stimuli.

Considering the transient and plastic nature of macrophages, paired with the negative prognosis of intra-tumoral M2 macrophage accumulation, several approaches have been developed to repolarize M2 macrophages to an M1 phenotype. Macrophages, much like T cells, also have immune checkpoints. The prevention of tumors from activating innate immune checkpoints, is another approach in preventing the suppression of macrophage anti-tumor responses. Alternative approaches that manipulate the plasticity of macrophages are being heavily explored. Several of these strategies are described in the following sections.

### Pro-Inflammatory Stimulation *via* TLR Agonism

The activation of TLRs, surface or endosomal proteins able to detect cellular damage and induce a proinflammatory immune response, have been broadly used therapeutically to alter macrophage activation in several diseases, including cancer (156–158) (**Figure 2**). The rationale is that the stimulation of these receptors, particularly within the tumor environment, may activate the pro-inflammatory response seen during the early stages of wound healing and infection, leading to the eradication of tumor cells (159, 160). Moreover, the release of tumor-derived DAMPs and neo-antigens during this process should generate a positive feedback loop to further increase the anti-tumor response (75, 159). A potential drawback of this form of therapy is tolerization, a state of unresponsiveness that appears after repetitive exposure to the same inductor, characterized by the release of anti-inflammatory factors that mask TLR activation (161).

Components of pathogenic organisms, such as LPS, derived mainly from *Eschericia coli*, are commonly used tools to activate macrophages and induce a pro-inflammatory state, often in combination with IFNγ to maximize the effects (162). However, LPS administration in humans produces severe toxicity and multiple exposures rapidly lead to tolerance, thus new strategies to improve its clinical use are currently being investigated (162). More recently, TLR3, TLR7/8 and TLR9 agonists have risen as new therapeutic alternatives to induce a TLR-dependent, tumor-localized pro-inflammatory response (163). For instance, the TLR7 agonist, Imiquimod, induces a robust rejection of skin primary malignancies and metastases by generating a pro-inflammatory tumor microenvironment in human patients (164) (**Table 1**). Similarly, polyinosinic-polycytidylic acid (poly-IC), a TLR3 agonist, triggers T cell tumor infiltration and Th1 responses, which should in turn activate macrophages through IFNγ signaling, to reduce malignant growth (165). Finally, the TLR9 agonist family CpG oligodeoxynucleotides (CpG ODN) have also shown strong cancer cytotoxic effects by exerting a potent tumor-localized immunostimulatory action (166) (**Table 1**). Based on early successes, these TLR agonists are currently in Phase 1/2/3 clinical trials (162, 163).

To target macrophages more specifically, nanoparticles that take advantage of the phagocytic properties of macrophages are being developed. After injection, nanoparticles are trafficked to the tumor where they are engulfed by macrophages. Techniques are being developed to package TLR agonists into nanoparticles for more specific activation of these immune cells (167). This novel approach would reduce the off-target effects of TLR agonists on other immune cells, such as lymphocytes, as well as to reduce their tolerizing effects (168). Furthermore, injected nanoparticles tend to accumulate in the tumor because of often ill-formed and leaky tumor vasculature, leading to a therapy more targeted to intra-tumoral macrophages (169). Loading β-cyclodextrin nanoparticles with the TLR7/8 agonist R484 has surfaced as one of the most promising techniques to restrain tumor growth by shifting TAM behavior to the M1 state (170).

### Activating ATP NOD-Like Receptors to Promote M1 Polarization

Purinergic activation of macrophages plays a crucial role for the secretion of the pro-inflammatory cytokines, IL-1β and IL-18, and can be mediated through the activation of the NLRP3 inflammasome (171–173) (**Figure 2**). Cellular stress (e.g., exposure to chemotherapeutics, toxins, and radiation) and tissue damage are key contributors to ATP release into the extracellular environment (174). Release of ATP is one of the most potent DAMPs for immune activation, promoting M1 macrophage polarization and increasing macrophage tumoricidal potential (87, 129, 175), (**Figure 2**). However, to maintain the cellular ATP equilibrium, tumor cells, macrophages, and other immune cells, express ectonucleotidases to maintain the concentration gradient. CD39 and CD73 are ectonucleotidases that are involved in the formation of the metabolite adenosine (ADO). CD39 sequentially hydrolyzes ATP and ADP to form AMP, whereas CD73 hydrolyzes AMP to form ADO (**Figure 2**). This shift in the concentration gradient also acts as a switch to a more M2-like functional program and attenuates the anti-tumor response. Adenosine activates ADO/purinergic G-coupled protein receptors on tumor and immune cells, such as macrophages, to induce immunosuppression (176). Likewise, ADO also functions to inhibit TLR signaling and the secretion of proinflammatory cytokines such as TNFα, IL-6, and IL-8 from activated human monocytes (177). Given the contrasting nature of ATP versus ADO signaling for macrophage activation in tumor immunity, this interface serves as a potential target for the clearance of tumor cells. Inhibition of CD39 in preclinical models have shown significant promise in diminishing the immunosuppressive activity of TAMs, whereas inhibition of CD73 proved effective in controlling metastatic growth (178) (**Table 1**). Furthermore, combinational therapeutic strategies employing innate immune checkpoint inhibitors and anti-CD39 or anti-CD73 promoted antitumor immunity (88). Lastly, antagonism of the ATP receptors (P2X7) increases tumor infiltrating immune effector populations and decreases tumor burden (130) (**Table 1**).

### Macrophage Polarization by Targeting Intracellular Signaling Mechanisms

In addition to mimicking extracellular pro-inflammatory stimuli, intracellular signaling pathways are also being targeted to reduce the prevalence of M2 signaling in tumors. This has been observed in the tumor-mediated manipulation of macrophage PI3Kγ signaling to reduce the pro-inflammatory response (179). Actually, targeting PI3Kγ pharmacologically has effectively “flipped the switch” from M2 to M1 in preclinical models (179, 180). PI3K is a family of phosphorylation enzymes that act on the 3’ end of phosphatidylinositol (PI) and work in conjunction with the Akt family of serine/threonine kinases and the mechanistic target of rapamycin complex (mTORC) 2 to switch the activation status of TLR-stimulated macrophages to a less pro-inflammatory program (181, 182) (**Figure 2**). PI3K/Akt signaling is involved in migration and diapedesis of innate immune effectors such as neutrophils and monocytes/macrophages and is associated with the upregulation and stabilization of hypoxia-induced transcription factors in macrophages (183). Induction of these transcription factors is associated with the hypoxic tumor microenvironment and stimulates M2-like characteristics in macrophages, thus supporting tumorigenesis and metastasis (184–186). Moreover, the PI3K/Akt pathway also promotes macrophage-mediated remodeling of the ECM, angiogenesis and immunosuppression of the adaptive immune response. Inhibition of PI3K signaling has shown considerable effects in regulating VEGF expression, a known factor that stimulates the adoption of the M2 functional program (183). There are several preclinical and clinical studies aimed at manipulating PI3K signaling to improve tumor outcomes. Inhibition of this pathway has been shown to increase macrophage infiltration and production of proinflammatory cytokines and chemokines (187). Akt signaling has differential downstream effects and deficiencies in Akt1 induced M1 activation (188). Consequently, inhibition of Akt signaling disrupts mTORC2 aggregation which diminished macrophage viability and proliferation (189).

The signal transducer and activator of transcription (STAT) signaling pathway is also of clinical interest. Downstream of several receptor tyrosine kinases, the STAT family communicates signals from the cytosolic face of the plasma membrane to the nucleus, where STAT dimers act as transcription factors and transcriptional modulators. STAT1 is recognized as a pro-inflammatory mediator and signaling can be initiated by type I and II interferons, growth factors, TLR activity and cytokine release. STAT1 signaling has broad effects on cancer and can either be antitumoral or pro-tumoral. Antitumoral STAT1 signaling is usually attributed to the tumoricidal activity of M1 macrophages while the pro-tumoral action is affiliated with the enrichment of STAT1-dependent genes that protect against genotoxic damage or promote tumor growth (190). Conversely, STAT3 is broadly recognized as an anti-inflammatory regulator, stimulating M2-like macrophage polarization. STAT3 phosphorylation can be triggered by interleukins such as IL-8, IL-10, IL-35 and growth factors such as EGF. Following activation, STAT3 signaling promotes a myriad of pro-tumoral outcomes such as the inhibition of apoptosis, cell proliferation, metastasis, angiogenesis and therapeutic resistance (41, 191). Studies targeting the activation of STAT1 or the suppression of STAT3 may be crucial for manipulating the balance of M1/M2 signaling.

Other transcription factors are also under study for potential roles in M1/M2 plasticity. These include KLF6, Zeb1 and NFAT1. KLF6 is a transcriptional regulator of macrophage polarization that serves as a phenotypic switch to transform M2-polarized TAMs to M1, effectively inhibiting tumor proliferation and migration (192). Contrariwise, ZEB1 is associated with TAM pro-tumoral activity, indicated by its ability to pioneer epithelial to mesenchymal transition to maintain tumor progression and initiate metastasis (8). Nuclear factor of activated T cell (NFAT) also supports the M2-like phenotype of TAMs through the regulation of interleukins (IL-6, IL-10, IL-12) and multiple TLR-induced genes such as iNOS (193). NFAT1 is overexpressed in TAMs and promotes tumor cell proliferation, invasion and metastasis and facilitates the recruitment of macrophage populations that are associated with poorer outcomes (194, 195). Given the role of NFAT signaling in regulating immune homeostasis, NFAT inhibition may effectively suppress anti-inflammatory cytokine production while subsequently initiating pro-inflammatory and tumoricidal programs within these tumor-associated macrophage populations.

Unfortunately, because individual transcription factors tend to be involved in transcriptional regulation throughout the genome, specifically targeting them to selectively target individual regulatory programs remains elusive. However, as time goes on, it may be possible to more selectively target individual immune cell types or add co-factors to increase specificity, yielding more robust anti-tumor efficacy.

### Manipulating Macrophage Metabolism to Increase M1 Polarization

The metabolic changes associated with M1/M2 polarization may also regulate activation state (196, 197). Much like the distinct glutaminase-dependent differentiations of Th17 and Th1 T cells to regulate the immune response (198), direct metabolic changes in macrophages, or the output of altered metabolism, can affect M1/M2 polarization.

Arginase is essential for amino acid metabolism and has potent immunomodulatory effects through the catalysis of L-arginine. L-arginine is involved in nitric oxide synthesis which contributes to the tumoricidal activity of macrophages (199). However, the catabolism of L-arginine by arginase results in the formation of L-ornithine and its decomposition product, putrescine, which are known to support the cell growth and proliferation of tumor cells (199–202). Furthermore, increased production by TAMs impairs the antitumor immune response (203). Likewise, putrescine induces macrophage efferocytosis to prevent inflammation and promote tissue repair (204), a hallmark of tumor progression. Catabolism of L-arginine also has devastating consequences for other immune effectors, such as cell cycle arrest and anergy (203). Inhibition of arginase I expression reduces tumor burden and subsequently increases lymphocyte infiltration within the tumor microenvironment (205, 206) indicating significant potential for clinical testing.

Like arginase, indoleamine 2,3-dioxygenase (IDO1) is an immunosuppressive molecule secreted by TAMs. IDO1 catabolizes tryptophan to kynurenine which binds to the aryl hydrocarbon receptor to trigger a myriad of immunoregulatory mechanisms in immune cells (207). The signaling cascade triggered by IDO1 enzymatic activity facilitates immune evasion by diminishing lymphocyte responsiveness and anticancer immunosurveillance (208–210). IDO1 activity is also suggested to increase tolerance in macrophages, downregulate antigen presentation molecules (HLA-DR) and decreased macrophage phagocytic activity (211). Furthermore, IDO has also been shown to increase M2 polarization and recruitment while inhibition of IDO activity increases M1 populations (212). IDO1 inhibition prevents tryptophan depletion and subsequently blocks the associated downstream immunosuppressive signals (213, 214). This suggests that targeting IDO enzymatic activity in tumors that overexpress this enzyme may improve macrophage polarization to M1, immune activation and immunotherapeutic efficacy.

### Targeting Innate Immune Checkpoints to Improve Therapeutic Outcomes

Much like the adaptive immune response, immune checkpoints have been discovered and characterized for innate immune cells. One example is the Tyro3/Axl/Mer (TAM) family of receptor tyrosine kinases, (**Figure 2**). During normal physiological processes, this family of receptors is instrumental in apoptotic cell engulfment and degradation (efferocytosis). The TAM family of receptors has 5 known ligands, Gas6 (215), Pros1 (216), Gal3 (217), Tubby and Tulp1 (218). As cells undergo apoptosis, phosphatidylserine that has flipped from the cytosolic face of the plasma membrane to the extracellular region is recognized by these ligands to form a bridge to the TAM receptors. However, these ligands can also activate the TAM receptors in the absence of phosphatidylserine (219), though activation is reduced. Lastly, kinase inhibition or genetic loss of Mer prevents internalization of apoptotic material (220, 221).

In addition to its role in efferocytosis, genetic lack of Mer is associated with hypersensitivity to TLR activation (222, 223), suggesting its role in limiting the innate immune response and preventing autoimmunity. More recently, it was shown by Lemke and Rothlin, in dendritic cells, that activation of Mer initiates an anti-inflammatory program involving upregulation of Socs1/2 (224). Later, Cook et al., demonstrated, in the context of cancer, that genetic deletion of Mer was associated with reduced M2 macrophage polarization with increased M1 (225). Ubil et al. later showed that tumor-secreted Pros1, acting on Mer and Tyro3 induces the downregulation of pro-inflammatory gene expression (51). Mice bearing tumors with genetic deletion of Pros1 showed increased intra-tumoral macrophages that were skewed towards the M1 phenotype. This was associated with increased adaptive immune infiltrate with approximately 5-fold more CD4+ and CD8+ T cells as well as a ~50% reduction in Tregs. Mice with Pros1 deficient tumors lived ~30% longer than mice with parental tumors. Furthermore, addition of the TLR7/8 agonist, Resiquimod, did not improve survival in mice bearing Pros1 replete tumors whereas survival duration was doubled for mice whose tumors lacked Pros1. Taken together, these findings demonstrate that tumor secretions can dampen the innate, macrophage, response and subsequently the adaptive immune response. TAM kinase inhibitors are currently in Phase I clinical trials for the treatment of human cancers.

Another marker involved in immune checkpoints and expressed by intra-tumoral macrophages is PD-L1. PD-L1 is generally associated with expression by tumors, particularly in response to IFNγ. When tumor expressed PD-L1 binds to PD-1 on T cells, it leads to T cell inactivation and facilitates tumor immune evasion. Tumors are also able to induce expression of PD-L1 in macrophages to similarly limit the action of effector T cells (226). Macrophage PD-L1 - T cell PD-1 interactions are, therefore, at the interface of innate and adaptive immune responses.

Several PD-1 and PD-L1 targeted therapeutics are currently in the clinic for treatment of various forms of cancer (227). In addition to the direct effects of blocking PD-1/PD-L1 interactions, PD-1 targeted treatments also induce secondary effects, such as the increased polarization of macrophages from a pro-wound healing phenotype to a more anti-tumor, pro-inflammatory, state. Xiong et al. characterized intra-tumoral macrophage polarization states of MC38 tumor bearing mice after anti-PD-1 treatment. They observed an increase in the numbers of M1-like and M1/M2 intermediate macrophages with a decrease in M2-like phenotypes. Using IFNγ depletion of supernatants from tumors which had either been treated with vehicle or anti-PD-1 antibody, they determined that IFNγ was a primary driver of macrophage polarization (228). Presumably, anti-PD-1 treatment of tumor bearing mice led to increased T cell activation, including IFNγ secretion. In turn, polarization of intra-tumoral macrophages were skewed towards an M1 state, including increased antigen presentation and expression of pro-inflammatory cytokines. Activated M1 macrophages increased T cell activation in a self-reinforcing cycle, ultimately leading to reduced tumor growth. This study succinctly demonstrates the importance and inter-relatedness of the innate and adaptive immune functions in limiting tumor progression.

### Targeting “Don’t Eat Me” Signaling to Improve Macrophage Activation and Antitumor Immunity

A crucial aspect of macrophage activity is phagocytosis, the internalization of cells, pathogens, and other particles for tissue homeostasis. As key endocytosing immune cells, macrophages are the primary phagocytic population and should be able to recognize aberrant cells and clear them using this process. However, tumor cells express anti-phagocytic ligands or “don’t eat me” signals similar to healthy cells in order to avoid elimination.

CD47 is an immunoglobulin that is crucial in self recognition for the maintenance of immune tolerance and homeostasis. It complexes with the signal regulatory protein α (SIRPα) on phagocytic cells to inhibit uptake and subsequent immune activation (229). However, this molecule is also expressed on the surface of many tumor cells and plays a key role in immune evasion (**Figure 2**). CD47/SIRPα signaling leads to the phosphorylation of the SIRPα cytoplasmic immunoreceptor tyrosine-based inhibition motifs (ITIM) resulting in the recruitment of the tyrosine phosphatases SHP1/2. This signaling mechanism prevents the accumulation of myosin at the phagocytic synapse, effectively inhibiting phagocytosis (230–232). This process is crucial in preventing uncontrolled clearance of healthy cells but becomes a detriment based on its role in facilitating immune evasion in cancer. As such, these signals are also targeted to improve the antitumor response. CD47 blockade has shown significant efficacy in the treatment of several hematological cancers and solid tumors which may be mediated by innate immune effector populations such as macrophages (94, 95, 233, 234) (**Table 1**). Furthermore, preclinical models of the CD47/SIRPα signaling axis are highly efficacious for treating multiple cancer types and are currently being probed in clinical trials.

CD24 is another “don’t eat me” signal that is expressed by many tumor types (**Figure 2**). CD24 is a glycosylphosphatidylinositol anchored protein that is known to complex with Siglec10 on macrophages and other innate immune cells for the suppression of the inflammatory response in many conditions including sepsis, liver damage and infection (85, 235, 236). Like CD47 signaling, the CD24/Siglec10 signaling axis results in the recruitment of SHP1/2 at the ITIMs of Siglec10, inhibiting the TLR-mediated inflammatory response and the cytoskeleton rearrangement required for phagocytosis (85). As such, the CD24/Siglec10 complex is a potent inhibitor of macrophage phagocytic activity and is protective of cancer cells. Inhibition of the CD24/Siglec10 signaling axis restores the macrophage-mediated antitumor response by enhancing phagocytic clearance of tumor cells (85, 86). Moreover, increased uptake of antigenic materials is also associated with increased immune activation and infiltration within the tumor microenvironment (85).

The importance of these signaling cascades in regulating macrophage plasticity are extensively studied and new models are currently being probed to increase innate immune activation and improve current immunotherapeutic approaches. A summary of these targets and their effect on macrophage activity within the tumor microenvironment, along with their development status, are described in **Table 1**.

## Current Experimental Modeling of M1/M2 Phenotypes May Not Accurately Represent Intra-Tumoral Macrophage Polarization States

To model macrophage responses, the M1/M2 paradigm was developed and dates back more than 20 years (237). In early models, naïve macrophages were induced to adopt two known polarization states (238). Since then, through decades of research, multiple *in vitro* models of M1 and M2 polarization have been developed in which various exogenous stimuli can induce activation states that mimic physiological conditions (e.g., pathogenic infection (239–241), pro-inflammatory activation by T cells (242, 243), etc.). At present, experimental macrophage models have been delineated into 5 core subsets: M1, M2a, M2b, M2c and M2d (244), (**Figure 1**).

Historically, activation of the M1 state has been modeled using stimuli such as LPS, IFNγ (a pro-inflammatory signal derived from activated T cells) or both in combination. While LPS induces TLR4 activation and downstream NFκB signaling, IFNγ binds the IFNgR1/2 complex, leading to STAT1 phosphorylation and nuclear translocation to mediate pro-inflammatory gene expression (245, 246). Alternatively, addition of TNFα (247) to naïve macrophages yields a similar activation state. TNFα binds to TNFR1 and TNFR2, leading to activation of downstream signaling cascades including p38 (248, 249) and others (250–253). The pro-inflammatory signaling pathways tend to converge on NFκB, STAT1 and MAPK pathways, with significant crosstalk effectively leading to similar outcomes in terms of gene expression changes and activation states.

M2 activation states are comparatively more complicated with at least 4 different subsets being identified, including M2a, M2b, M2c and the relatively newer M2d phenotype (152, 254, 255) (**Figure 1**). Induced by IL-4, IL-13 or the combination thereof, M2a has been described as an anti-inflammatory and pro-wound healing subset (256–258). M2b, which is induced by addition of IL-1β, has shown immuno-regulatory properties and associated gene expression (244, 259). M2c macrophages, induced by treatment with IL-10, show increased expression of immune suppressive and tissue remodeling markers (260). Some indications also suggest efferocytosis is increased in M2c macrophages (261). Finally, in an attempt to create a model of TAMs (M2d), it was discovered that treatment with IL-6 could cause upregulation of tumor growth and angiogenesis markers (262).

At this point, there is not one clearly prevailing macrophage M2 subset that best represents tumor associated macrophages. Instead, researchers often combine multiple stimuli, such as IL-4 (M2a), IL-13 (M2a) and IL-10 (M2c), which are present in the tumor microenvironment, to mimic tumor associated macrophages (263, 264).

While continually improving, our understanding of intra-tumoral macrophage activation states have led to an iterative improvement in models. However, newer and better methodologies are currently being utilized to disaggregate our current population-level understanding. Specifically, single cell RNA-seq (sc-RNA-seq) has refined our understanding of intra-tumoral macrophage heterogeneity and called into question some of our existing paradigms on “either/or” M1/M2 polarization.

## Single-Cell RNA-Seq Data Sheds New Light on Intra-Tumoral Macrophage Polarization

Based on established *in vitro* models of macrophage polarization (M1/M2), early characterization of intra-tumoral macrophages focused on a few pro-inflammatory or pro-wound healing markers (e.g., iNOS, IL-1, CD206, etc.) to identify activation states. As more nuanced models of polarization have been developed, additional markers have been identified, demonstrating that rather than adhering to distinct polarized types, macrophages exhibit a spectrum of overlapping activation states. Further complicating the ability to describe tumor associated macrophages is that spatial location and microenvironmental factors can have major impacts on polarity, causing macrophages in one part of the tumor to have very different activation states than those in adjacent locations. The advent of single cell RNA-seq has opened new venues for understanding intra-tumoral macrophage activation and may identify misconceptions about how macrophages behave in the tumor microenvironment. This new technique allows for the characterization of individual cells within the tumor resident immune cell subset. Depending on the process flow, immune cell subtypes may be enriched prior to single-cell RNA-seq analysis (265, 266) or bioinformatically identified based on expression patterns (267). Several variations of single-cell RNA-seq exist, some of which also incorporate locational data.

### Characterization of Macrophage Activation State in Tumors

Using single-cell RNA-seq to characterize immune subset in primary breast cancer samples, Chung et al. found that macrophages tend toward the M2 phenotype (265), confirming previous findings that breast cancer tends to foster M2 polarization (46, 268). Of the 515 cells from 11 patients characterized, most non-carcinoma cells in the cancer samples were identified as immune cells based on their gene expression signatures. TAMs were primarily found to have pro-wound healing M2-associated profiles (269, 270). A key finding of this paper is that it supports the notion that in breast cancer, many macrophages and other innate and adaptive cell populations have an immune suppressive phenotype.

Recognizing that there is robust heterogeneity of intra-tumoral macrophage polarization states, single cell RNA-seq is also being used to determine whether there are discrete activation states or whether there is a contiguous spectrum driven by local microenvironmental conditions. Azizi et al., employed a large-scale, high-dimensional analysis platform to characterize the immune profiles of more than 45,000 cells from eight breast carcinomas, matched with normal breast tissue, blood and lymph nodes using single-cell RNA-seq (271). To do so, they collected CD45 positive cells from treatment-naïve breast cancer patients including estrogen receptor (ER+) and progesterone receptor (PR+) positive, human epidermal growth factor receptor 2 amplified (HER2+) and triple negative (TNBC) tumors. These CD45+ cells were isolated by fluorescence-activated cell sorting (FACS) and subjected to single-cell RNA-seq using the inDrop platform (272, 273). Data was preprocessed using the SEQC pipeline with the Bayesian clustering and normalization method, Biscuit, utilized for data analysis. One of the key findings of the study is that intra-tumoral macrophages have higher numbers, diversity and activation relative to those derived from normal tissues or lymph nodes. Somewhat surprisingly, the authors of this study found a positive correlation between M1 and M2 gene expression, with simultaneous co-expression of markers associated with both activation states. This is in direct contrast to previous results from *in vitro* model studies, in which one or more agents used to activate macrophages led to one aggregate activation state, either M1 or M2.

A different study, characterizing the heterogeneity of macrophages activation states in gliomas using single-cell RNA-seq made a similar observation on the simultaneous co-expression of M1 and M2 markers in TAMs. This study, conducted by Muller et al. (274), compared marker expression of two macrophage populations – brain-resident microglia, derived from progenitors that migrated to the central nervous system (CNS) and bone marrow-derived monocytes that extravasate through the blood brain barrier and differentiate into macrophages. Similar to Azizi et al., Muller et al., found that macrophages could co-express M1 and M2 markers simultaneously with 66% of tumor associated macrophages co-expressing the canonical M2 marker, IL-10, while also expressing the M1 marker, TNFα. They confirmed their results by using flow cytometry of tumor derived macrophages to show that CD11b+ cells could co-express the M1 co-stimulatory marker, CD86, while also expressing CD206.

Taken together, these studies call the M1/M2 polarization paradigm into question. While, to some extent, supporting the notion that a spectrum of intra-tumoral macrophage activation states exist (275, 276), the finding of simultaneous M1 and M2 associated markers by macrophages is quite novel. Perhaps historical use of conventional models coupled with aggregate analyses of pooled macrophage populations fail to detect a more widespread phenomenon of M1/M2 marker co-expression in tumors. Further experiments and analysis will be required to confirm these finding. Also, development of model systems that better recapitulate the dual activation states observed *in vivo* may yield better understanding of how intra-tumoral macrophages will respond to targeted therapeutics. Perhaps most importantly, these findings suggest that activating, or re-activating, the M1 phenotype in tumors may consequently lead to concurrent increased M2 polarization, thereby confounding outcomes.

### Using Single Cell RNA-Seq Based Methods to Characterize Macrophage Activation While Incorporating Spatial Localization Within the Tumor

Conventional large-scale characterization of macrophage polarization loses spatial resolution. As such, novel single-cell RNA-seq/bioinformatic approaches are being developed that provide contextual identity. One such technique involves the use of spatial transcriptomics (277). This method performs unbiased mapping of transcripts over entire tissue sections using spatially barcoded oligo-deoxythymidine microarrays. Individual microarray spots capture transcriptome information from between 10-200 cells and the data is integrated with single cell RNA-seq data to provide both cellular context and transcription data at the single cell level. Using this approach, Moncada and colleagues performed multimodal intersection analysis on patient pancreatic ductal adenocarcinoma (PDAC) tumors (278). One of their key findings was that macrophages seem to adhere to the M1/M2 paradigm and exist in two main subpopulations. The first was a pro-inflammatory M1 subset, which expressed IL-1β, and a second subset, which expressed M2 associated genes like CD163 (278). Likewise, the two subpopulations were differentially localized, with M1 macrophages enriched in the cancerous regions or the stroma, while M2-like macrophages were enriched in the ducts. This data demonstrates that two opposing macrophage polarizations can exist in the same tumor, though their activation state is driven by local micro-environmental conditions. These findings suggest that, fundamentally, treatments may be more effective if they can be selectively targeted to regions where they will make the biggest change. Conversely, systemic treatment with an M1 inducing agent could disrupt essential processes and induce off-target effects.

### Derivation of M2 Macrophage Subpopulations

Circulating monocytes are recruited to tumors by the expression of chemoattractants such as CCL2 (279–281), S100A8 and S100A9 (282, 283). Once monocytes extravasate, they are thought to differentiate into M1 or M2 macrophages based on signals from the tumor microenvironment. In a recent study, Song et al. used single-cell RNA-seq to characterize the differentiation process of extravasating monocytes. 11,485 cells from Non-Small Cell Lung Cancer (NSCLC) patients were used to develop a model of divergent monocyte differentiation into M1 or M2 macrophages. While there were differences between patients, on average, a substantially larger proportion of the recruited monocytes adopted the M2 phenotype (283). In CD14+ cells derived from in NSCLC samples, expression of polarization markers was stratified along a continuum effectively providing a snapshot of macrophage differentiation states. Work by Song et al., may enable the identification of specific lineage markers that will allow prediction of future differentiation states. They also identified signals from tumor-derived epithelial cells that skew differentiation to the M2 phenotype. By better understanding the process through which tumor resident M2 macrophages are derived, it may be possible to develop specific interventions that prevent accumulation of M2 macrophages.

## Open Questions in Macrophage Plasticity During Cancer

Macrophages are a highly plastic innate immune cell subset. Depending on contextual cues from their local environment, they adopt phenotypes across a spectrum of activation states, ranging from pro-inflammatory (M1) to pro-wound healing (M2). Further, macrophages, both individually and in aggregate, can readily transition from one polarization state to the next depending on the most recent signals prevailing in their environment. This plasticity allows them to effectively adapt to the changing environments associated with infection and wound healing and facilitate the return to immune homeostasis. Unfortunately, in the context of cancer, macrophage plasticity is subverted to benefit continued tumor progression. Either by tumor-mediated suppression of M1 polarization or through the evolved lack of pro-inflammatory cues associated with cancer, intra-tumoral macrophages are generally of the pro-wound healing (M2) phenotype. The pro-wound healing properties which would be beneficial during injury repair, such as production of growth factors or promotion of angiogenesis, support continued tumor cell proliferation and tumor expansion.

Recognizing the inherent plasticity of macrophages, several therapeutics have been developed to either reduce the number of intra-tumoral macrophages, thereby reducing the M2 pool, or alter the M1/M2 balance to favor a more pro-inflammatory/anti-tumor response. Numerous clinical trials have demonstrated that increasing M1-associated polarization or effector functions can improve clinical outcomes. This is, perhaps, not surprising since a pro-inflammatory milieu is associated with better patient outcomes for many cancer types. However, to realize the promise of these new treatment modalities, several factors still need to be considered. As we have learned from adaptive immune targeted treatments, activation or checkpoint blockade alone are not likely to be sufficient to generate durable responses in several cancer types. Rather, macrophage targeted therapies will likely require co-treatments targeting the cancer directly (e.g., chemotherapy) or the adaptive immune response (e.g., checkpoint directed therapeutics) or both. Also, for the most part, M1 polarization is thought to reduce tumor growth. However, chronic and persistent local inflammatory conditions are also known to induce tumor formation (284–287). A prime example is that increased inflammation associated with obesity can actually increase the likelihood of tumor progression (288). Several other preclinical models of inflammation, such as colitis-induced colon cancer (72–76), have shown that persistent inflammation exacerbates tumor progression. As an illustration, in a high-fat diet induced inflammation model, prostate cancer progression was substantially increased (289). The rationale is that persistent cell damaging conditions may elicit genetic mutation or cell signaling alterations that foster tumor growth. While the current paradigm is that “more inflammation is better”, there is likely to be an optimal amount of inflammation so as not to induce secondary tumor formation.

Another key question to be addressed, in addition to finding optimal combinations, is how to limit potential engagement of the autoimmune response. Even if a macrophage targeted therapy is successful in generating an anti-tumor response, what are the best ways to ensure it is targeted strictly to the tumor and not surrounding healthy tissues or organ systems? While some delivery systems, like nanoparticles, favor intra-tumoral macrophages, many require systemic delivery, increasing the potential for off-target effects. Potentially compounding the likelihood of off-target effects is reliance upon the bystander effect to generate an anti-tumor response. For example, TLR agonists mimic PAMPs and DAMPs that would be released during infection or injury. However, the resulting immune activation does not target tumor-intrinsic moieties, but rather utilize the destructive potential of pro-inflammatory macrophages to either kill neighboring tumor cells or activate other local immune cells. This lack of tumor specificity opens the greater possibility of non-specific cellular damage or even autoimmunity based on the release of cryptic epitopes.

In addition to questions of developing targeted therapeutics, some basic scientific questions also remain unanswered about macrophages in the tumor environment. While several models have shown, *in vitro*, that macrophages can move from one polarization state to the next, it is unclear whether this is also true in tumors. For instance, lack of lineage tracing prevents the accurate monitoring of individual intra-tumoral macrophages to determine what happens after treatment. Are macrophages that are present in the tumor prior to treatment adopting another phenotype or is macrophage turnover the cause for an aggregate shift in polarization? Development and use of lineage tracing models would provide a more expansive knowledge of macrophage activation during treatment.

Other questions that have arisen with the advent of single-cell RNA-seq include whether there is a previously unknown macrophage state the possesses elements of both the diametrically opposed M1 and M2 phenotypes. Can both activation states co-exist in one cell or group of cells? What environmental or cell intrinsic factors would allow for dual expression of pro- and anti-inflammatory markers? Do these dual activation macrophages also exist during wound healing or response to pathogenic infection or are they a cancer-specific phenomenon? Are there ways in which these specialized cells can be modeled *in vitro*? Perhaps most importantly, how do pro-inflammatory inducing treatments affect dual M1/M2 macrophages? Does their presence confound treatments focusing on M1 induction? For instance, if a TLR agonist is utilized for treatment, does it also increase the expression of M2 associated markers, simultaneously activating and inactivating the immune response? Further analysis of single-cell RNA-seq data may answer these questions. However, it may be possible, using flow cytometry or other techniques, to isolate these cells and characterize them using more traditional biochemical methods.

While there is a more comprehensive understanding of macrophage biology now than in the past, development of macrophage targeted therapeutics has trailed behind those promoting the adaptive immune response. Continuing to address the unanswered questions presented here, as well continued testing, both alone and in combination with other therapeutics, may bridge the gap, providing new hope for improved survival of cancer patients.

## Author Contributions

TR - wrote the manuscript, prepared figures, and edited final work. NP-D - wrote manuscript and edited final work. PG - wrote manuscript. EU - conceptualized the work, wrote manuscript, and edited final work. All authors contributed to the article and approved the submitted version.

## Funding

NIH/NCI K22 Transition Career Development Award (1 K22 CA237742-01) - Funding for EU, and University of Alabama at Birmingham Development Funds - Funding for EU.

## Conflict of Interest

The authors declare that the research was conducted in the absence of any commercial or financial relationships that could be construed as a potential conflict of interest.
